# Supplementary material for: Programmable receptors enable bacterial biosensors to detect pathological biomarkers in clinical samples
Source: Nat Commun. 2021 Sep 1;12:5216. doi: 10.1038/s41467-021-25538-y (PMC8410942; doi:10.1038/s41467-021-25538-y)
Supplement: Supplementary file 1 — Supplementary Information [file 41467_2021_25538_MOESM1_ESM.pdf]

Supplementary materials for:

Programmable receptors enable bacterial biosensors to detect pathological biomarkers in clinical samples.

Hung-Ju Chang[1], Ana Zuniga[1], Ismael Conejero[1,2,3], Peter L. Voyvodic[1], Jerome Gracy[1], Elena Fajardo-Ruiz[1], Martin Cohen-Gonsaud[1], Guillaume Cambray[1], Georges-Philip Pageaux[4], Magdalena Meszaros[4], Lucy Meunier[4], and Jerome Bonnet[1*].

Affiliations:

[1]Centre de Biologie Structurale (CBS). INSERM U1054, CNRS UMR5048, University of Montpellier, France.

[2]Neuropsychiatry: Epidemiological and Clinical Research, Inserm Unit 1061, Montpellier, France.

[3]Department of Psychiatry, CHU Nimes, University of Montpellier, Montpellier, France.

[4]Department of Hepatogastroenterology, Hepatology and Liver Transplantation Unit, Saint Eloi Hospital, University of Montpellier, Montpellier, France.

* to whom correspondence should be addressed: jerome.bonnet@inserm.fr

These supplementary materials contain:

- Supplementary text.
- Supplementary figures S1 to S22.
- Supplementary tables 1 to 9.

Supplementary text.

NGS primers used in this study

I. 1st round PCR for adaptor and UMI barcode:

TcpP_Sensing_NGS_1st_Fw:
TCGTCGGCAGCGTCagatgtgtataagagacagNNNNNNNNCCAGAAACTTAGCGAGCAGAAG

TcpP_Sensing_NGS_1st_Rv:
GTCTCGTGGGCTCGGagatgtgtataagagacagNNNNNNNNgccacctgggatttccg

II. 2nd round PCR for index insertion:

P5_bc1	aatgatacggcgaccaccgagatctacacAACAACACtcgtcggcagcgtc
P5_bc2	aatgatacggcgaccaccgagatctacacAACGGTGGtcgtcggcagcgtc
P5_bc3	aatgatacggcgaccaccgagatctacacACCTTCTTtcgtcggcagcgtc
P5_bc4	aatgatacggcgaccaccgagatctacacATTGAGCCtcgtcggcagcgtc
P5_bc5	aatgatacggcgaccaccgagatctacacCCAGGAAGtcgtcggcagcgtc
P5_bc6	aatgatacggcgaccaccgagatctacacCGTCCAATtcgtcggcagcgtc

P7_bc1	caagcagaagacggcatacgagatCGTCCAATgtctcgtgggctcgg
P7_bc2	caagcagaagacggcatacgagatGTGTCGTTgtctcgtgggctcgg
P7_bc3	caagcagaagacggcatacgagatAACAACACgtctcgtgggctcgg
P7_bc4	caagcagaagacggcatacgagatACCTTCTTgtctcgtgggctcgg
P7_bc5	caagcagaagacggcatacgagatCCAGGAAGgtctcgtgggctcgg
P7_bc6	caagcagaagacggcatacgagatAACGGTGGgtctcgtgggctcgg

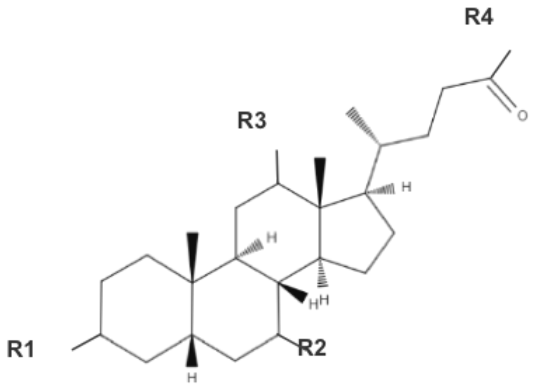

Name	Abbreviation	R1	R2	R3	R4
Cholic acid	CA	OH	OH	OH	OH
Glycocholic acid	GCA	OH	OH	OH	$NHCH_2COO-$
Taurocholic acid	TCA	OH	OH	OH	$NHCH_2CH_2SO_3-$
Chenodeoxycholic acid	CDCA	OH(α)	OH(α)	H	OH
Glycochenodeoxycholic acid	GCDCA	OH(α)	OH(α)	H	$NHCH_2COO-$
Taurochenodeoxycholic acid	TCDCA	OH(α)	OH(α)	H	$NHCH_2CH_2SO_3-$
Ursodeoxycholic acid	UDCA	OH(α)	OH(β)	H	OH
Glycoursodeoxycholic acid	GUDCA	OH(α)	OH(β)	H	$NHCH_2COO-$
Deoxycholic acid	DCA	OH	H	OH	OH
Glycodeoxycholic acid	GDCA	OH	H	OH	$NHCH_2COO-$
Taurodeoxycholic acid	TDCA	OH	H	OH	$NHCH_2CH_2SO_3-$
Lithocholic acid	LCA	OH	H	H	OH

Supplementary Figure 1. Chemical structures of different bile salts and corresponding abbreviation used in this study.

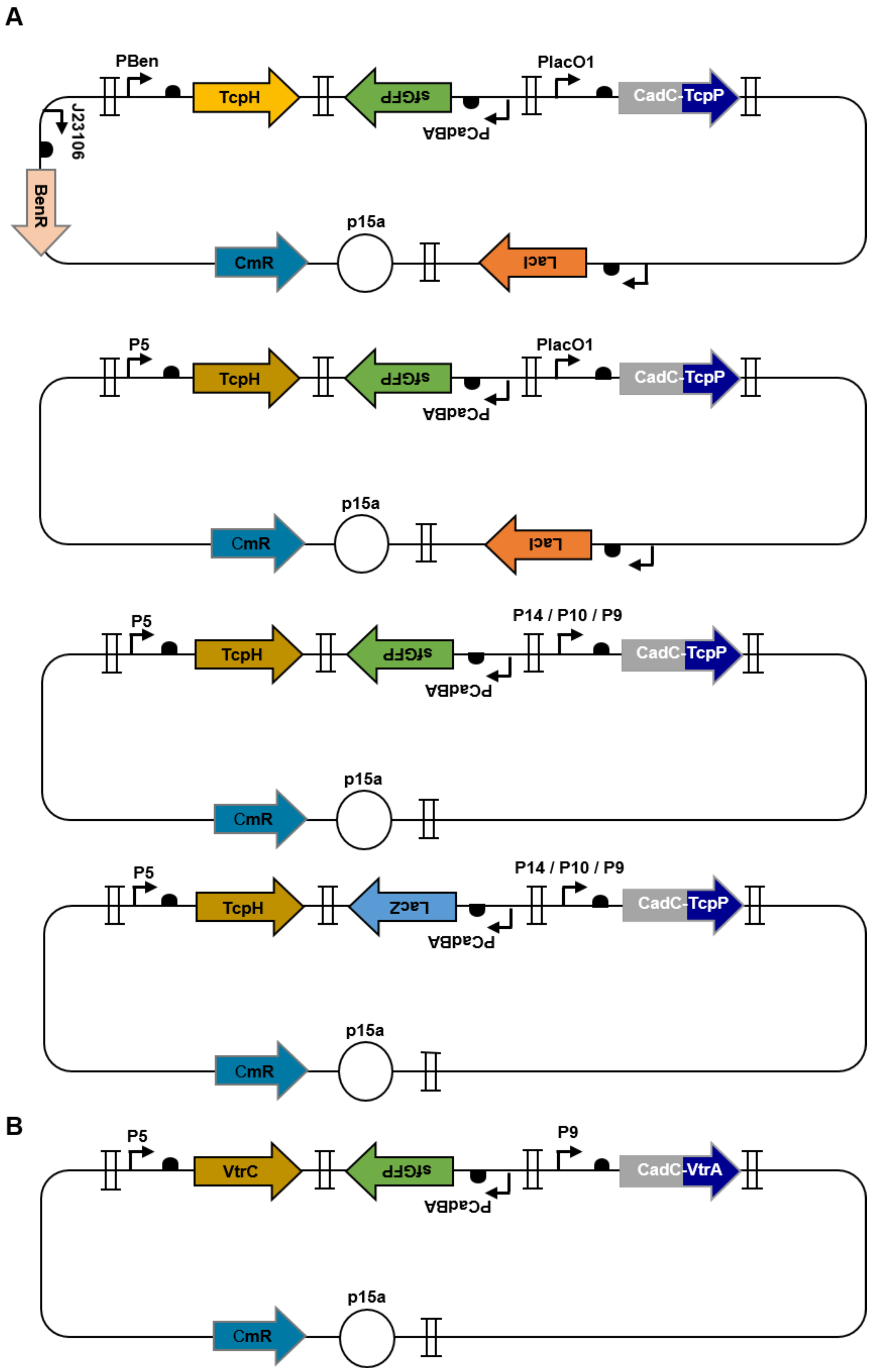

Supplementary Figure 2: Plasmid maps of (A) inducible, mixed type and constitutive CadC-TcpP/TcpH system; and (B) constitutive CadC-VtrA/VtrC system. *BenR*, the transcription factor BenR. *LacI*, the transcription factor LacI. CmR, the chloramphenicol resistance gene. Promoters are depicted by arrows; RBS are shown as solid half-circles; terminators are presented by Ⅱ symbols.

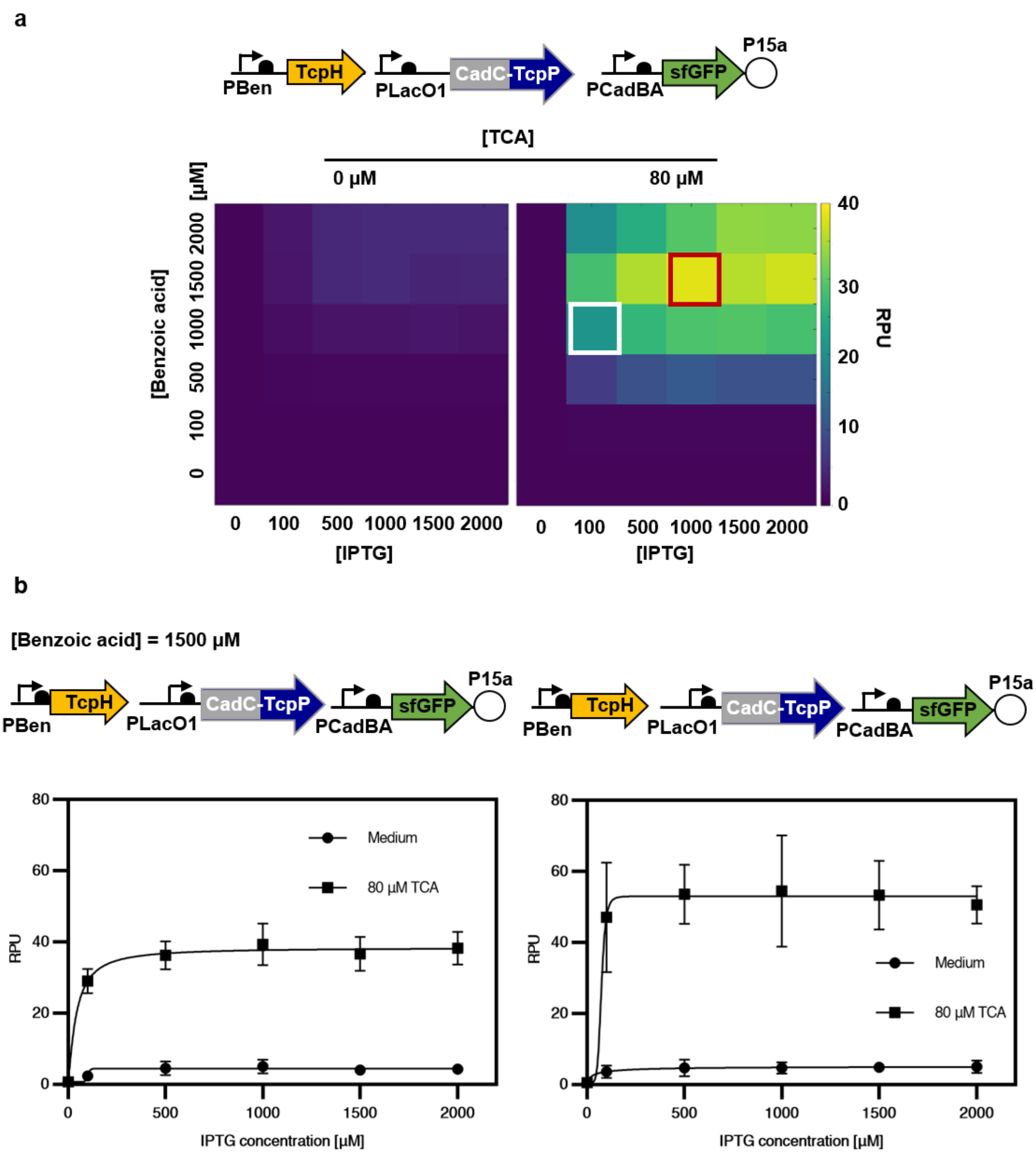

Supplementary Figure 3: System performance of inducible CadC-TcpP/TcpH system. (a) Response of inducible CadC-TcpP/TcpH system with or without the presence of ligand TCA at different expression levels induced by various concentrations of IPTG and benzoic acid. Schematic diagram of each genetic component and their corresponding control elements are listed above. RPU, reference promoter unit. The response with highest signal-to-noise ratio is highlighted with white square and the response with highest dynamic range is highlighted with red square. We found that there was an optimal expression ratio between the two proteins, i.e. maximizing protein expression did not lead to better output. (b) Comparing the different response between pLacO1-CadC-TcpP system with benzoic acid inducible (left panel) or constitutively expressed (right panel) TcpH, respectively. Inducible expression systems are useful to explore the parameter space of engineered systems. Yet the final bactosensor should operate using genes driven by optimal constitutive promoters. Constitutive expression reduces overall system complexity, DNA and metabolic footprint, and facilitates biosensor manipulation for real-world applications. Using constitutive promoters also helps avoiding toxicity or interference from the inducer molecule. Here we use the data from the inducible CadC-TcpP/TcpH system with highest dynamic range

(induced by 1500 μM benzoic acid, Fig. S2a) to compare with the data from PlacO1-CadC-TcpP with constitutively expressed TcpH. We first placed the TcpH gene under the control of the strong constitutive promoter P5[42], while keeping CadC-TcpP expression under IPTG control (right panel). The resulting combination showed a significant improvement in signal output level (swing) and signal-to-noise ratio compared to the system in which TcpH expression was under the control of the inducible pBEN promoter (left panel). These results suggested that the signal drop observed in supplementary Fig.3a at high benzoic acid concentration might be due to a deleterious effect of the inducer benzoic acid on the cellular physiology. The data points correspond to the mean value of three replicates performed in triplicate on three different days (n = 3 biologically independent samples). Error bars: ± SD. RPU: reference promoter units.

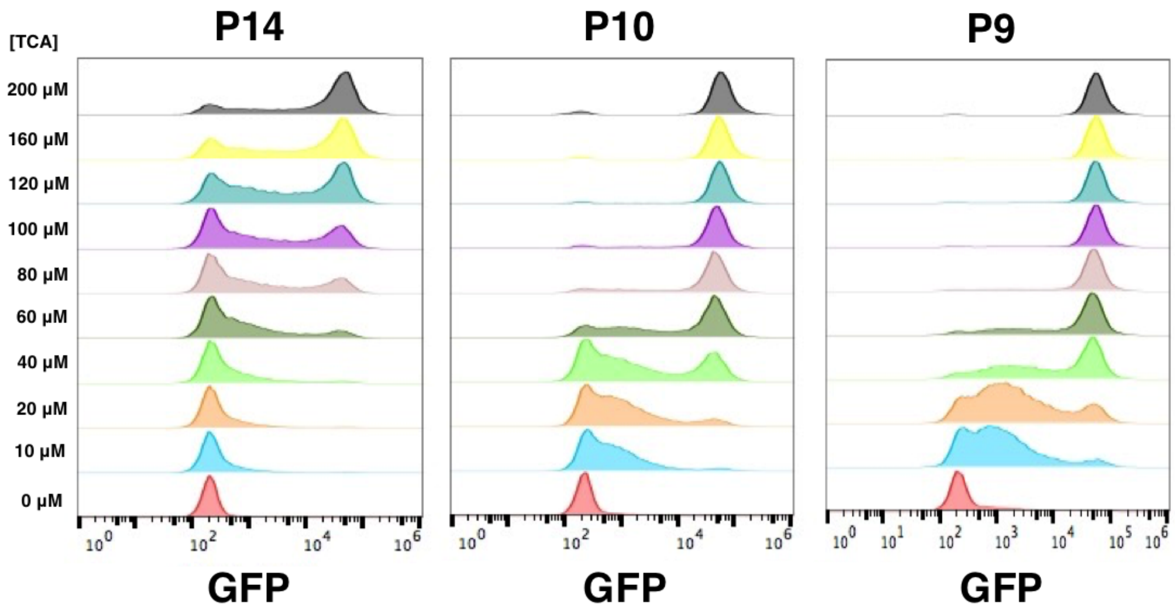

Supplementary Figure 4: Response of CadC-TcpP promoter variants (with P5_TcpH) to increasing concentration of ligand TCA. The TCA concentration is labeled at the left-y-axis. Bacteria cells were grown overnight, diluted 1:100 in 1 mL of LB with different concentrations of TCA and induced for 4 hours before flow cytometry analysis. Each histogram shows GFP fluorescence expressed as a result of TCA induction. A representative example is depicted here.

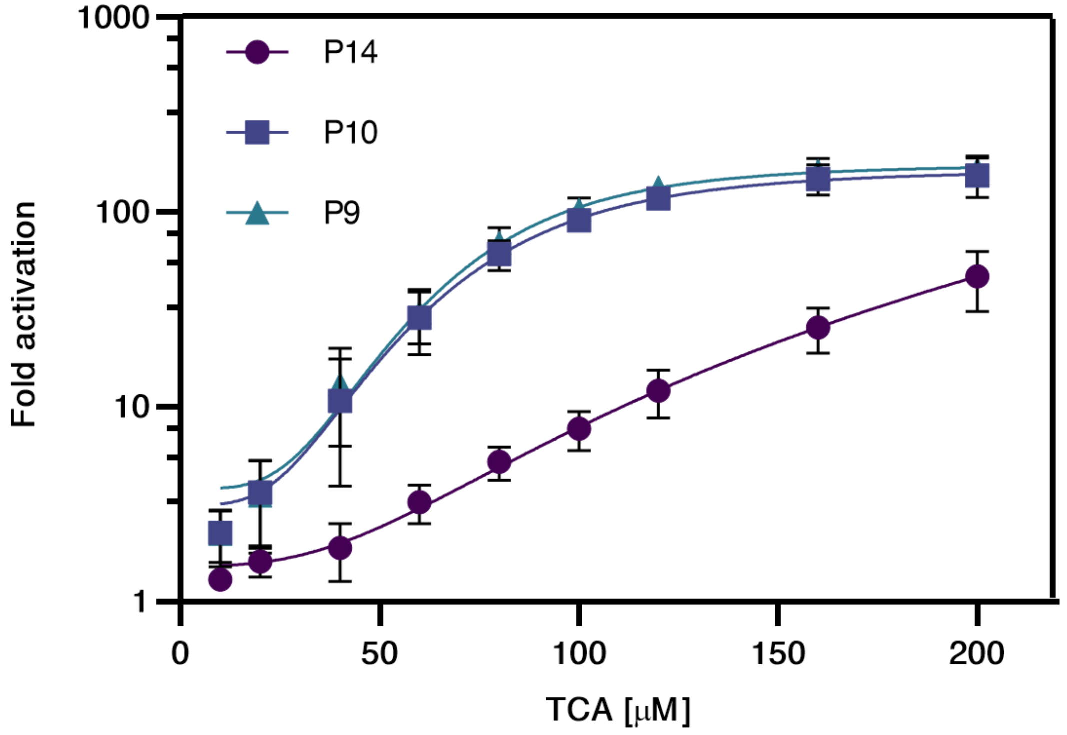

Supplementary Figure 5: Activation fold of CadC-TcpP promoter variants (with P5-TcpH) responded to different concentrations of ligand TCA. Bacteria cells were grown overnight, diluted 1:100 in 1 mL of LB with different concentrations of TCA and induced at 37°C for 4 h before flow cytometry analysis. The curves were fitted using a non-linear regression model with Hill Slope (four-parameter dose-response curve). Data points are the mean of three replicates performed in triplicate on three different days (n = 3 biologically independent samples). Error bars: ± SD.

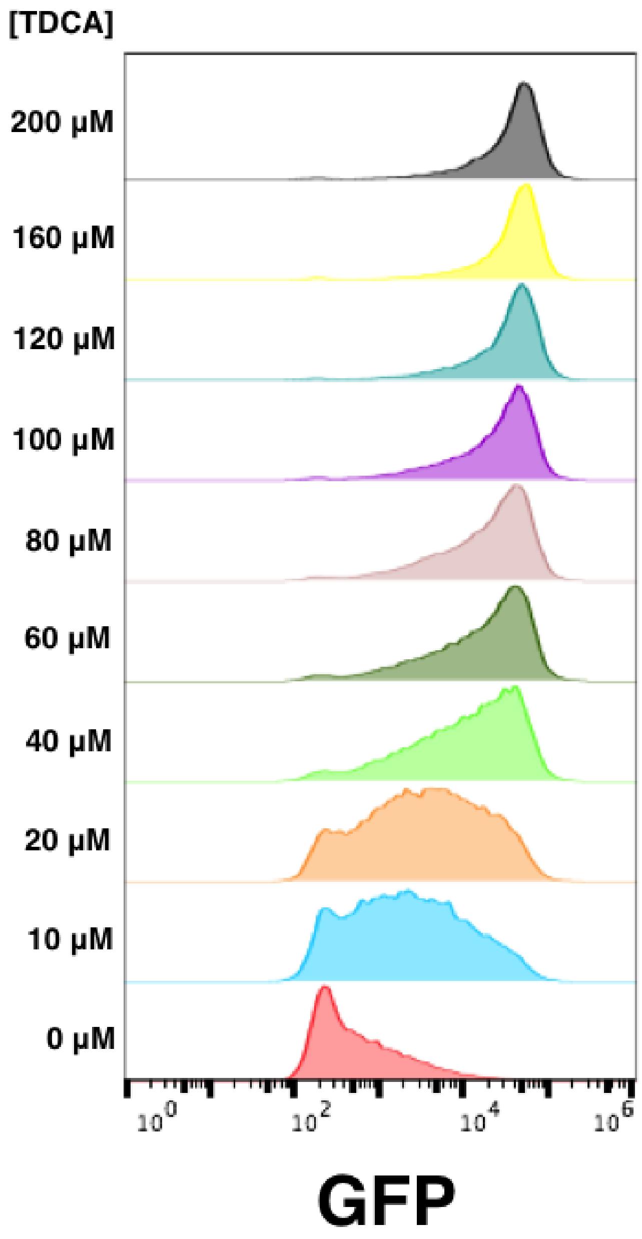

Supplementary Figure 6: Response of P9-CadC-VtrA_P5-VtrC to different concentrations of TDCA. Bacteria cells were grown overnight, diluted 1:250 and induced with different concentrations of TDCA in 1 mL of LB for 4 hours at 37 °C before flow cytometry analysis. Each histogram shows GFP fluorescence expressed as a result of TDCA induction. A representative example is depicted here. Different concentrations of TDCA used for titration are labeled at the left-y-axis.

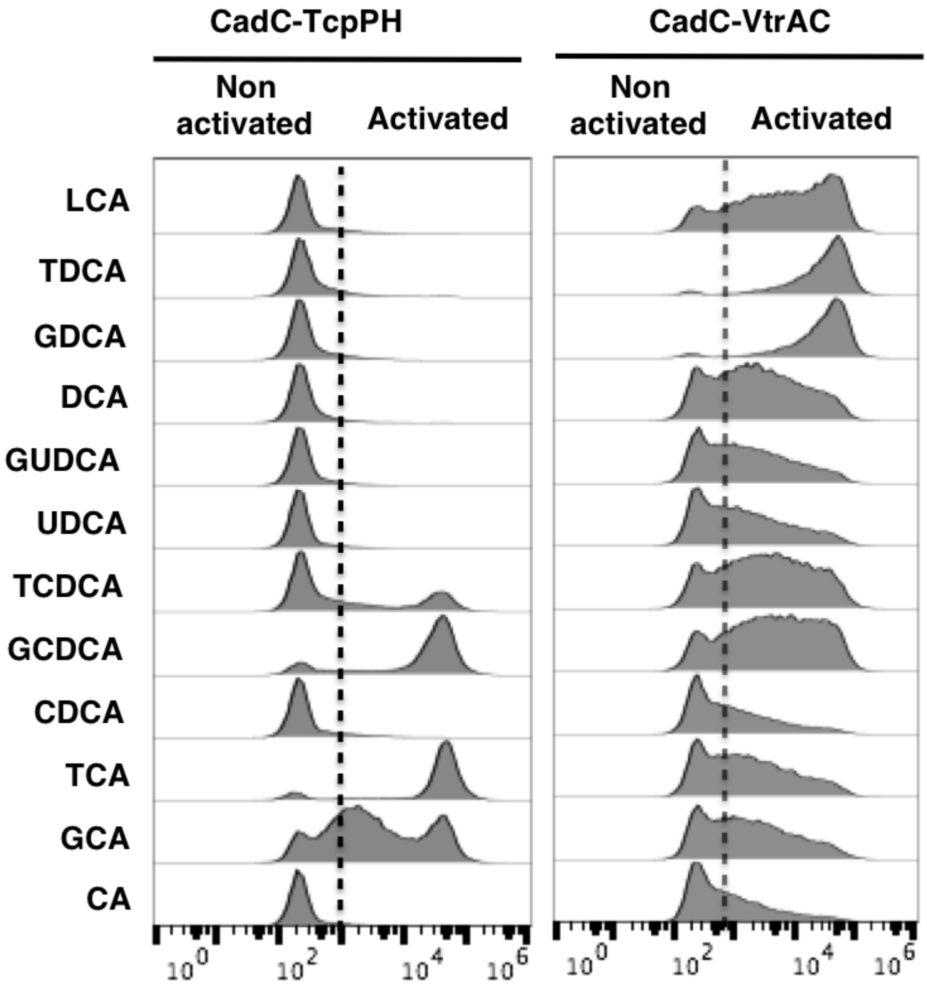

Supplementary Figure 7: Response of CadC-TcpPH and CadC-VtrAC system to different types of bile salts. The activated cells and non-activated cells are further distinguished by gating. The different bile salts used for profiling are labeled in the left-y-axis. Different from the TcpPH system which shows significant preference to primary conjugated bile salts, the VtrAC system shows different levels of preference to all bile salts. This result might reveal the potential of the hydrophobic inner chamber formed by VtrA/VtrC heterocomplex for the protein engineering of ligand specificity against different types of bile salts.

a

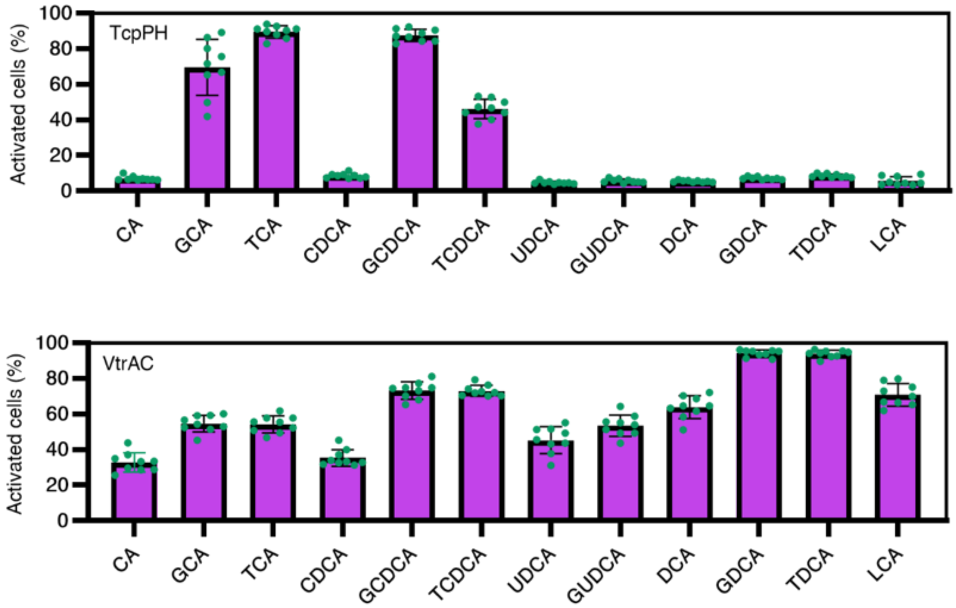

b

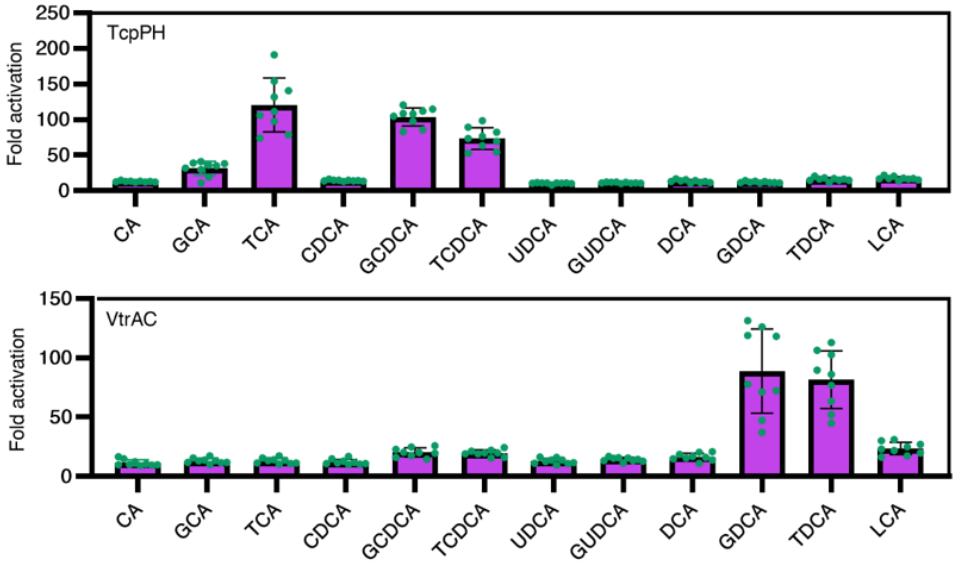

Supplementary Figure 8: Response of CadC-TcpPH and CadC-VtrAC system to different bile salts are presented as (a) percentage of activated cells and (b) fold activation. The population of activated cells are calculated through the gating shown in Fig. S7. The activation fold is calculated as the ratio of the geometric mean of activated cells divided by the geometric mean of non-activated cells. The bar graphs correspond to the mean value of three replicates performed in triplicate on three different days (n = 3 biologically independent samples). Error bars: ± SD. Cells growing in exponential phase were incubated with bile salts for 4 hours before flow cytometry measurement.

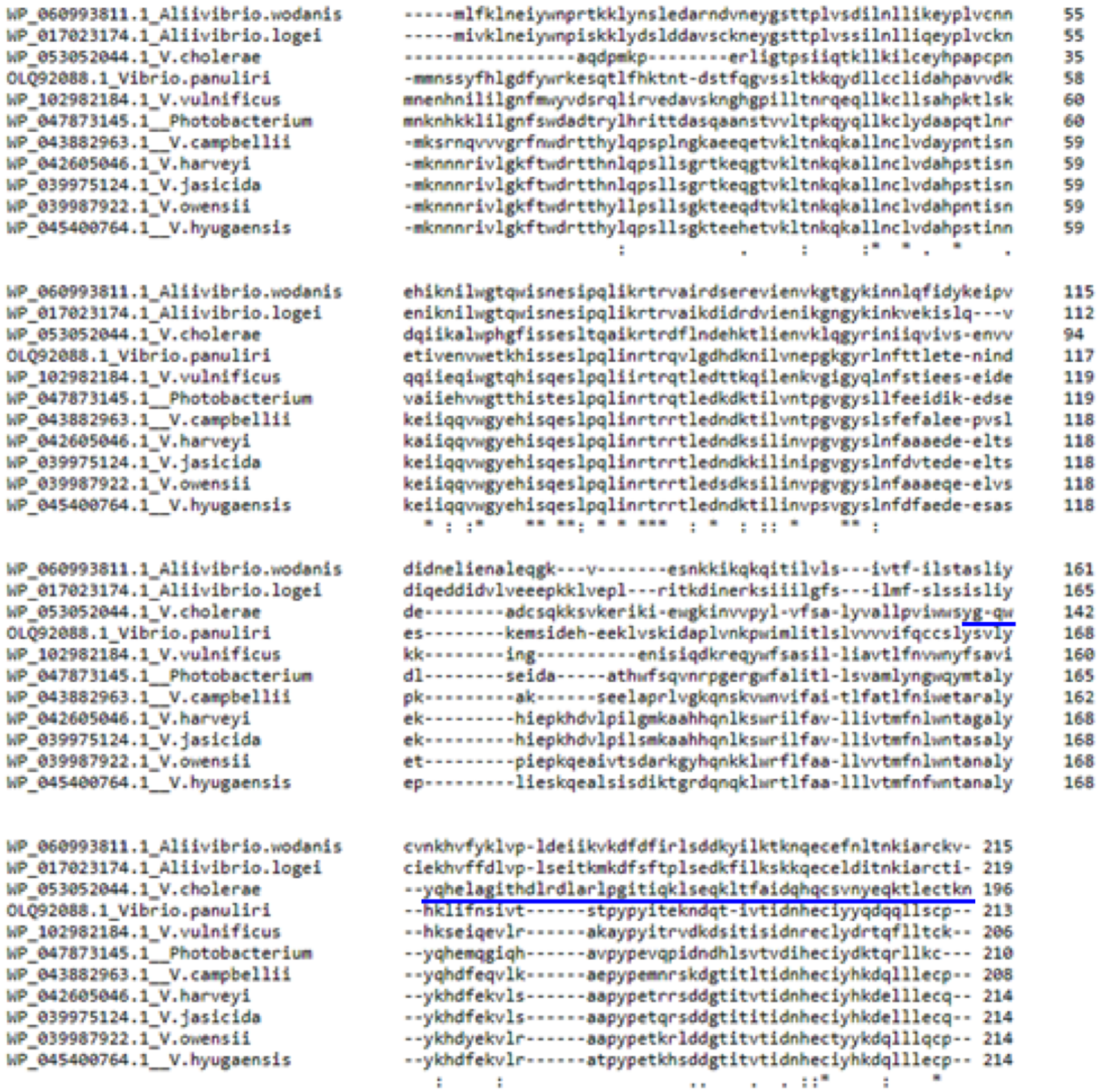

Supplementary Figure 9: Multiple sequence alignment of the periplasmic domains from TcpP homologous proteins. The periplasmic bile salt sensing domain of TcpP is highlighted by the blue line. TcpP homologs are found in *Vibrio* and *Aliivibrio* bacteria and their C-terminal periplasmic domains have high sequence similarities.

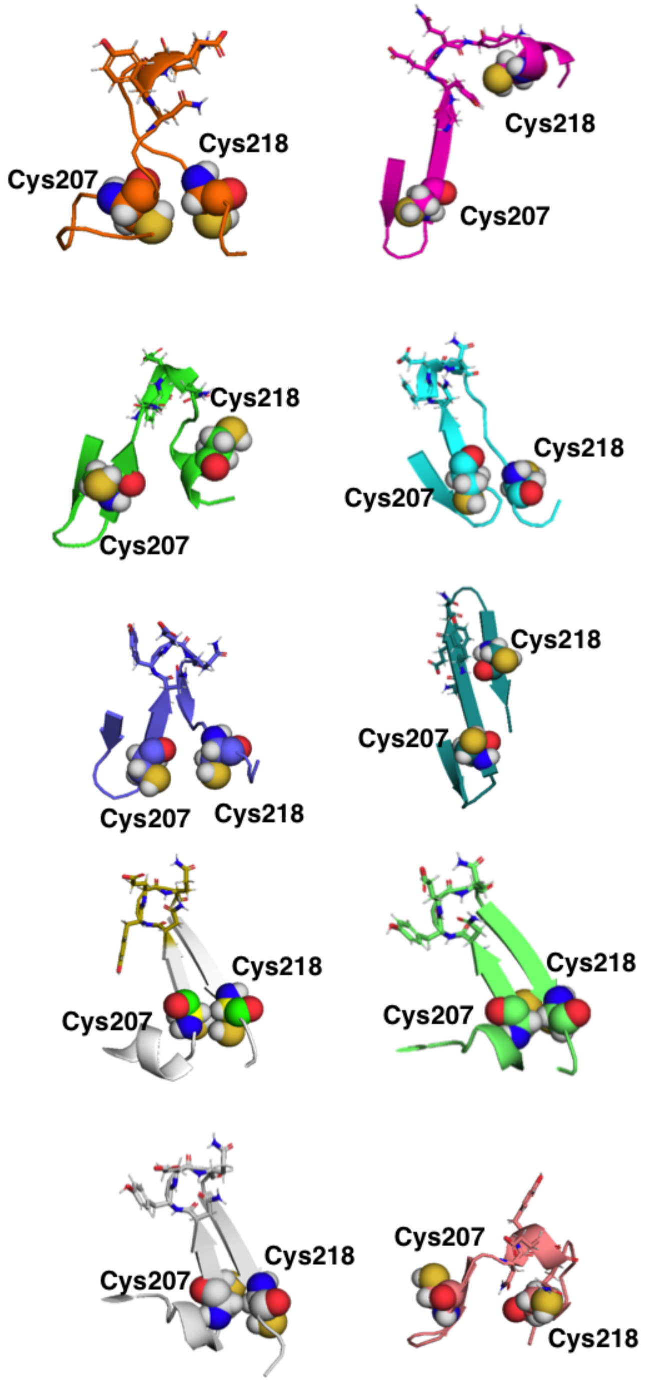

Supplementary Figure 10: Molecular modeling of TcpP periplasmic sensing domain. 10 different structural models of the TcpP C-term segment (residues 182 to 211) are presented here. The flexible loop region between Asn211 and Gln214 are labelled as sticks. The two cysteine residues are labelled as spheres, and show strong tendency to be close to each other in most of the models.

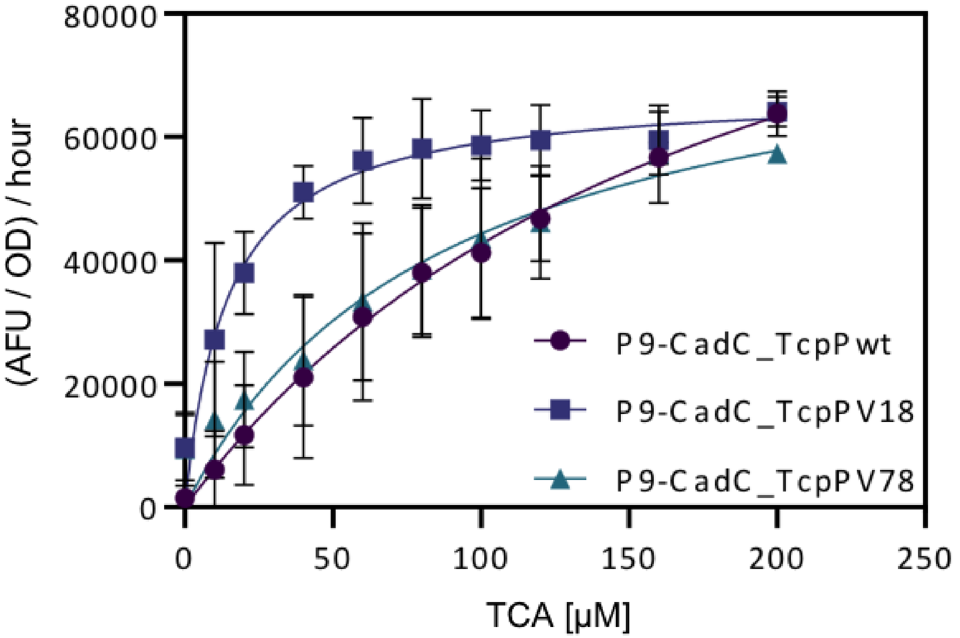

	TcpPwt	TcpPV18	TcpPV78
**Vmax**	123084	67529	83036
**Km**	188.8	14.44	87.4

Supplementary Figure 11: Kinetics analysis of CadC-TcpPwt and CadC-TcpP18. Bacteria cells were grown overnight and diluted 1:250 into 200µL 96-well plates with M9 minimal medium with 0.4% glycerol, plus different TCA concentration. Absorbance at 600nm and GFP (excitation 485 nm, emission 528 nm, gain 80) were measured every 10 minutes for 4 hours. Michaelis-Menten enzyme kinetics was evaluated by using GraphPad Prism (version 9.0.2) nonlinear regression model. The curve graphs correspond to the mean value of three replicates performed in triplicate on three different days (n = 3 biologically independent samples). Error bars: ± SD.

a

b

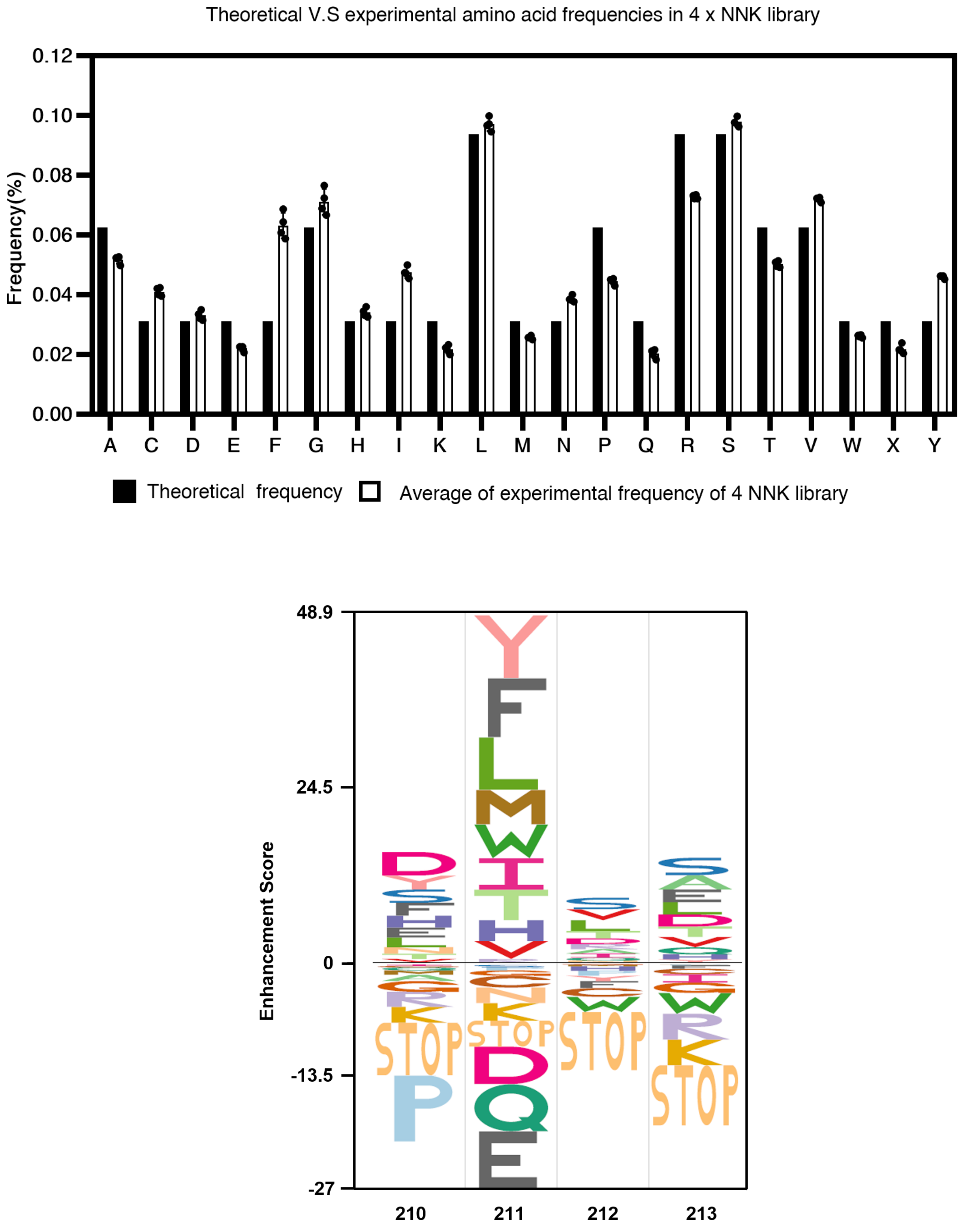

Supplementary Figure 12: (a) Comparison of theoretical and experimental amino acid frequencies in preselected NNK library. For verifying the quality of preselected TcpP synthesized 4 x NNK library, the summary counts of NGS results of each position were calculated as frequencies. The average values were further compared with theoretical frequencies of the NNK library. The bar graphs (experimental) correspond to the mean value of four sets of frequencies calculated from four different positions in the 4 x NNK library (n = 4 biologically independent samples). Error bars: ± SD. (b) The EDlogo plot of TcpP functional variants (from 3rd round of selection) compared with background frequencies (from preselected library) to highlight the enrichment and depletion score. The y-axis of the logo is the enhancement score of each amino acid. STOP: the 'TAG' stop codon in NNK degenerate codons.

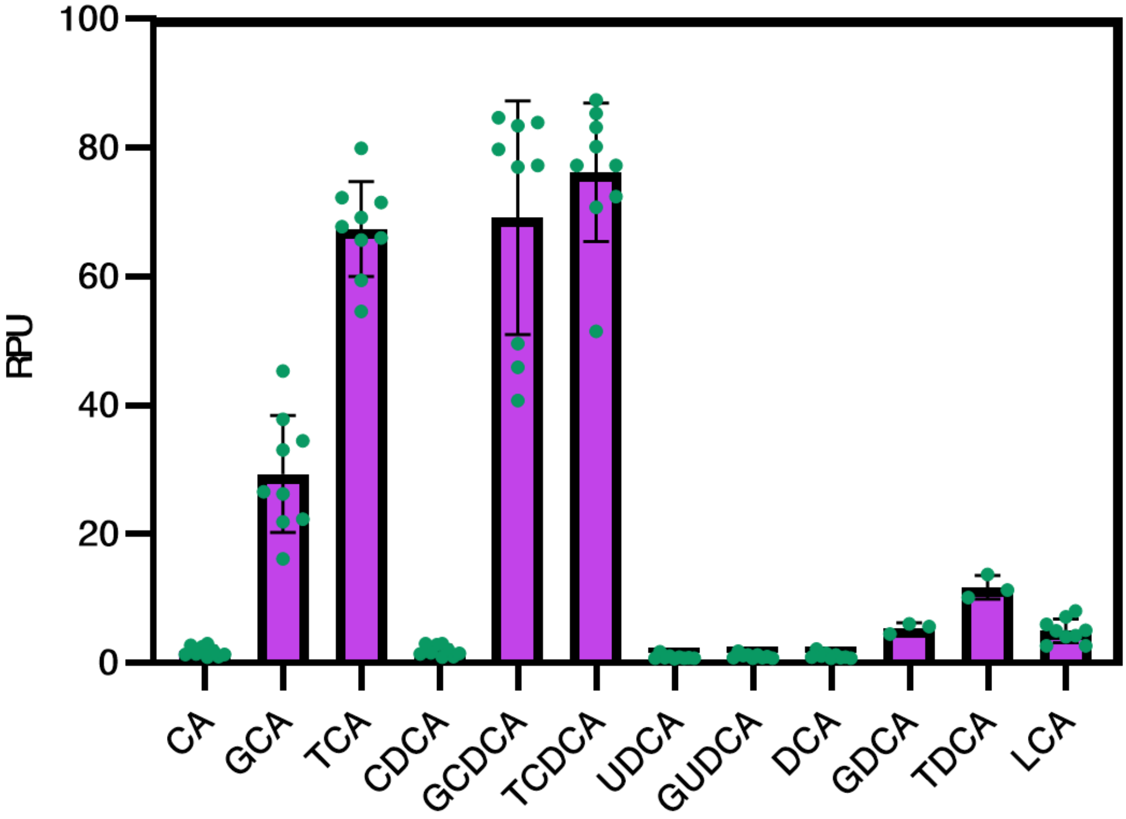

Supplementary Figure 13: Bile salt profile of TcpP18-GFP. Comparing the bile salt profiles among CadC-TcpP-sfGFP (Fig.2E), CadC-TcpP18-sfGFP (Fig.S13), and CadC-TcpP18-LacZ (Fig.4B), the bile salt profile differences between wild type TcpP and loop variant TcpP18 might be due to the alternation of ligand specificity (especially toward to secondary bile salts), rather than the alternation of output reporter gene or incubation methods. The bar graphs correspond to the mean value of three replicates performed in triplicate on three different days (n = 3 biologically independent samples, except for GDCA and TDCA were performed in one day for three replicates). Error bars: ± SD. RPU: reference promoter units.

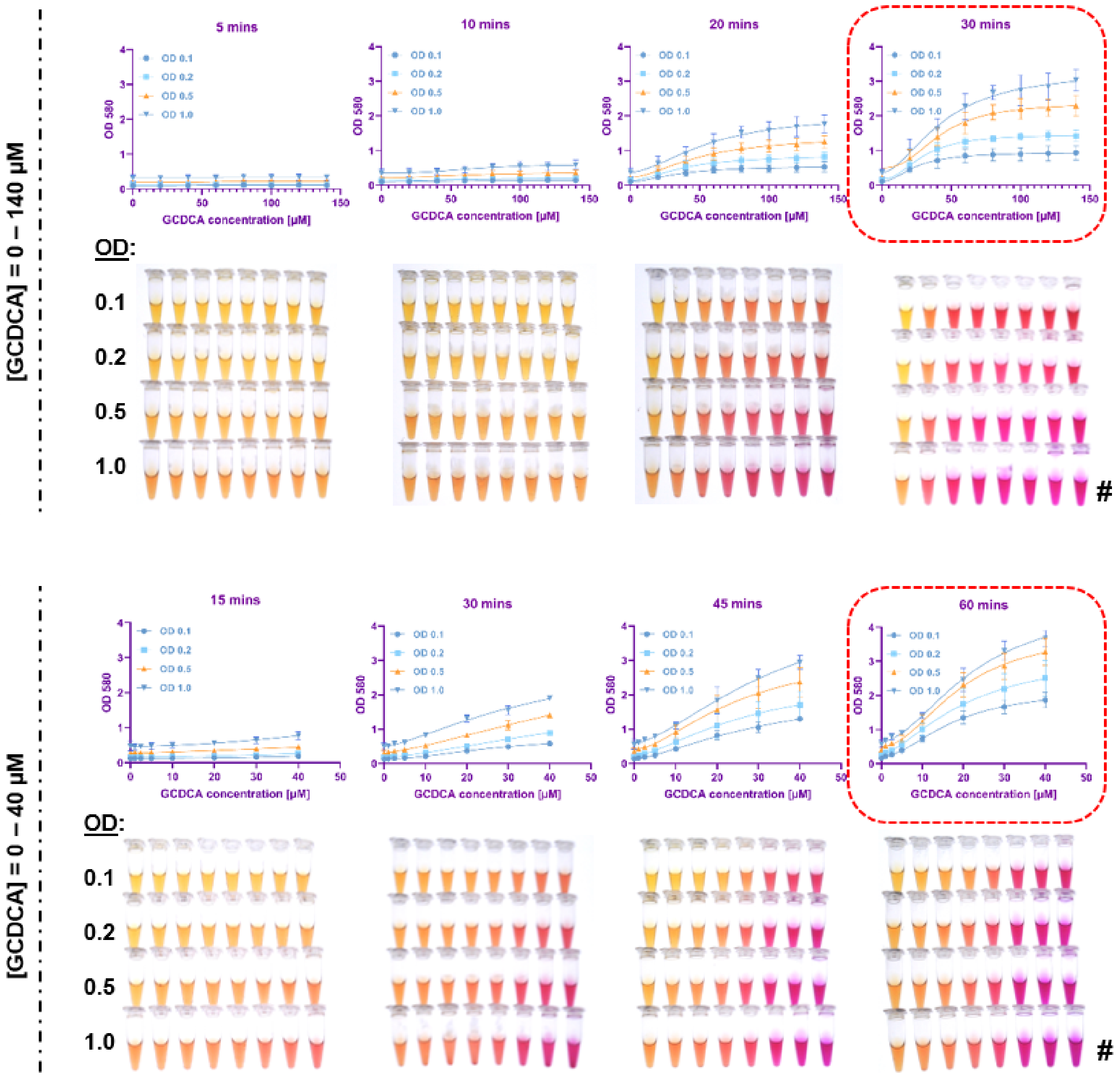

Supplementary Figure 14: Fine tuning system performance of TcpP18-LacZ to ligand GCDCA through cell density and sample incubation time. In order to optimize our system performance with significant visual differences or linear responses, the cell density and sample incubation time of TcpP18-LacZ were fine tuned and tested in two different concentration ranges of ligand GCDCA (0-140 μM, upper panel and 0-40 μM, bottom panel). Highlighted data set by red square correspond to the most significant signal swing and the hashtag (#) indicates its final reaction pictures. The curves here were fitted using a non-linear regression model with Hill Slope (four-parameter dose-response curve) using GraphPad Prism. The curve graphs correspond to the mean value of three replicates performed in triplicate on three different days (n = 3 biologically independent samples). Error bars: ± SD.

a

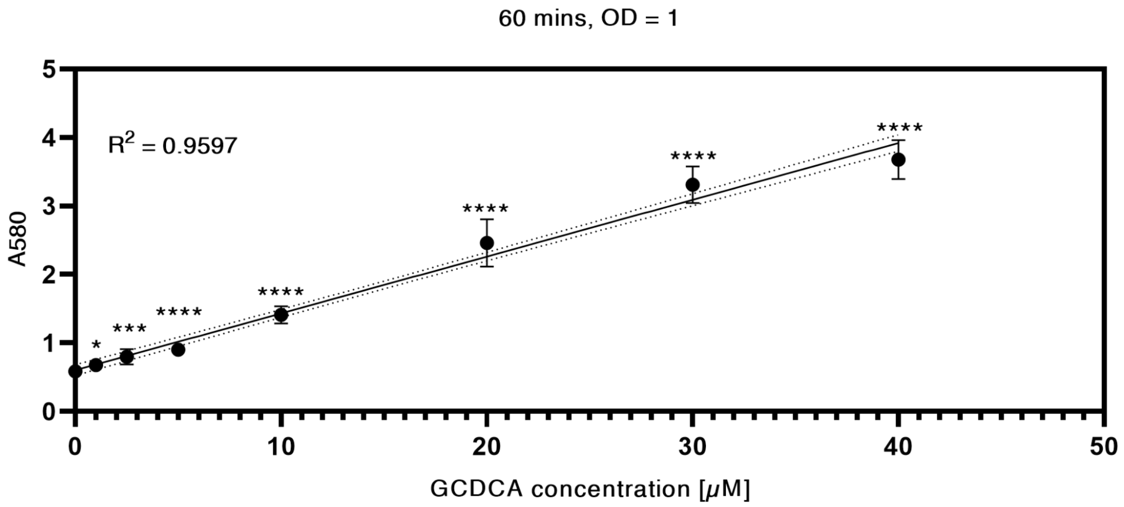

Supplementary Figure 15: Statistical analysis for the response of TcpP18-LacZ to 0 - 40 μM of GCDCA. In order to identify the reliable detection limit (which data point has significant difference against the background without ligand), and if the individual measurement is distinguishable from each other, here we use unpaired two tailed student t-test with Welch's correlation to analyze the statistical differences between different concentration of ligand GCDCA and the background with 9 data points (triplicates in three independent experiments). The incubation condition with the most significant linear response (cell density =1, incubation time = 60mins) is plotted here. The curve here was fitted using the linear regression model, using GraphPad Prism. The data here indicates that in proper incubation conditions, our bactosensor has good linear response to ligand GCDCA at 0 - 40 μM range. *p < 0.05, **p < 0.01, ***p<0.001, and ****p<0.0001, compared with signal without ligand GCDCA. The regions between two dash lines indicate 95% confidence bands of the best-fit line. Complete data analysis details (the exact P values) are available in the Source Data file. The curve graphs correspond to the mean value of three replicates performed in triplicate on three different days (n = 3 biologically independent samples). Error bars: ± SD.

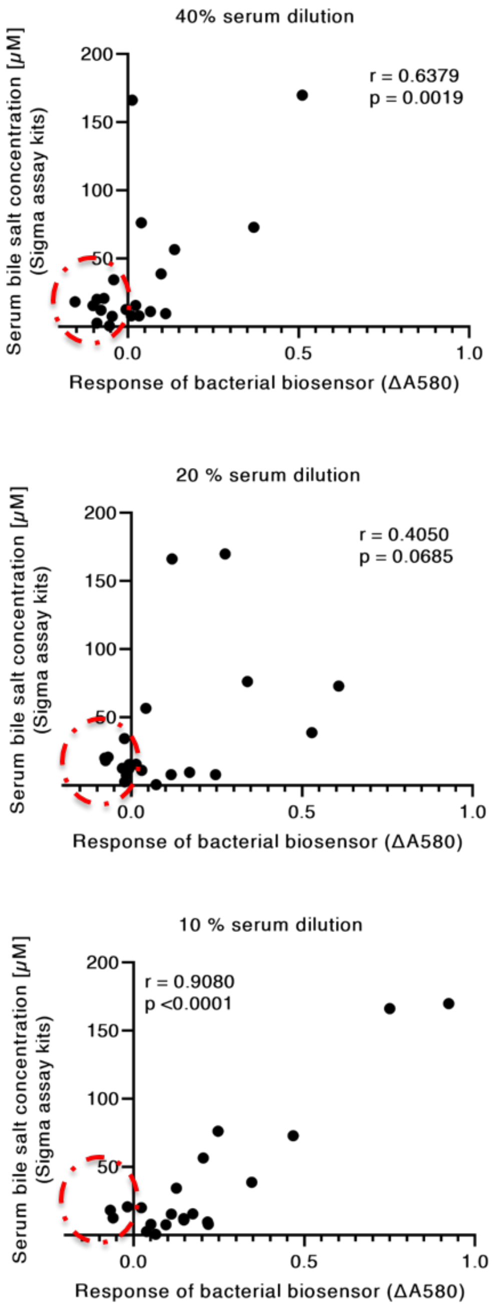

Supplementary Figure 16: Determination of the optimal serum dilution rate in fresh bacterial biosensor cells. In order to reduce the interference from serum to bactosensor assay, we tested the response of bactorsensors to 21 clinical serum samples at different dilution rates (20, 10, and 5 μL in final volume 50 μL with cells for final ratio as 40, 20 and 10%, respectively). With the increase of serum dilution rates, there are more positive responses (signal higher than background with only serum, ΔA580 >0) of bactosensor against different clinical serum samples: there are 10 samples have signal lower than background when the dilution ratio is 40%, and there are 3 samples have signal lower than background when the dilution ratio is 10% (red circles).

a

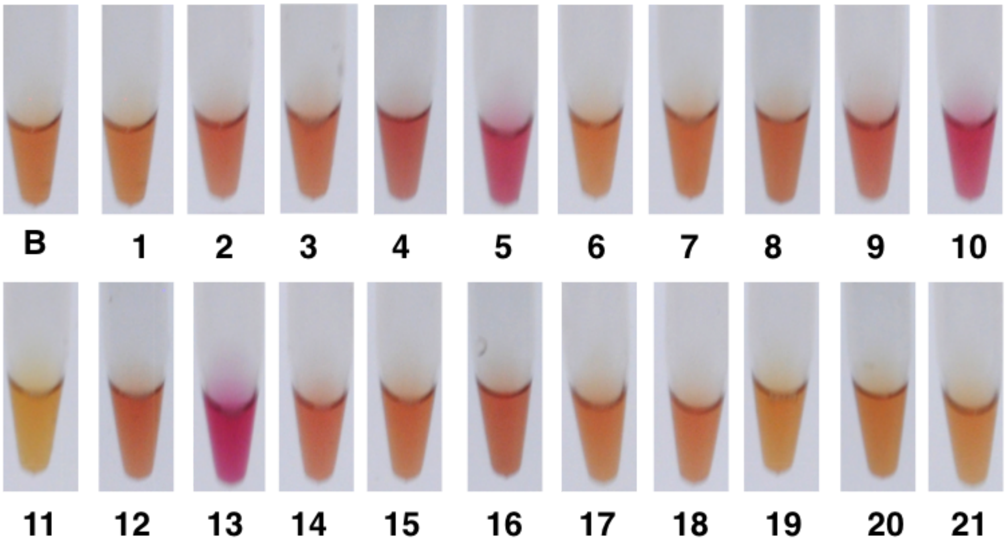

b

	Blank	P1	P2	P3	P4	P5	P6	P7	P8	P9	P10
A580	0.77	0.80	0.89	0.86	0.98	1.52	0.83	1.01	0.82	1.11	1.23
Δ A580		0.04	0.13	0.09	0.22	0.75	0.06	0.25	0.05	0.35	0.47
Sigma (μM)		2.56	34.44	7.59	7.86	166.21	0.62	76.22	7.86	38.74	72.91

	P11	P12	P13	P14	P15	P16	P17	P18	P19	P20	P21
A580	0.70	0.94	1.69	0.98	0.91	0.97	0.91	0.88	0.75	0.79	0.71
Δ A580	-0.07	0.17	0.92	0.22	0.15	0.20	0.15	0.11	-0.02	0.02	-0.06
Sigma (μM)	18.24	15.65	169.91	9.63	11.10	56.58	12.07	15.37	20.71	20.08	12.60

Supplementary Figure 17: Visible color changes of bactosensor in 10% clinical serum samples (a) and measured absorbance values for each patient (b). a. The original figures corresponding to Figure 5bB of 21 patients are presented here. b. The original data shown in Figure 5B and supplementary Figure 16 of bactosensor responses to 21 patients are presented here.

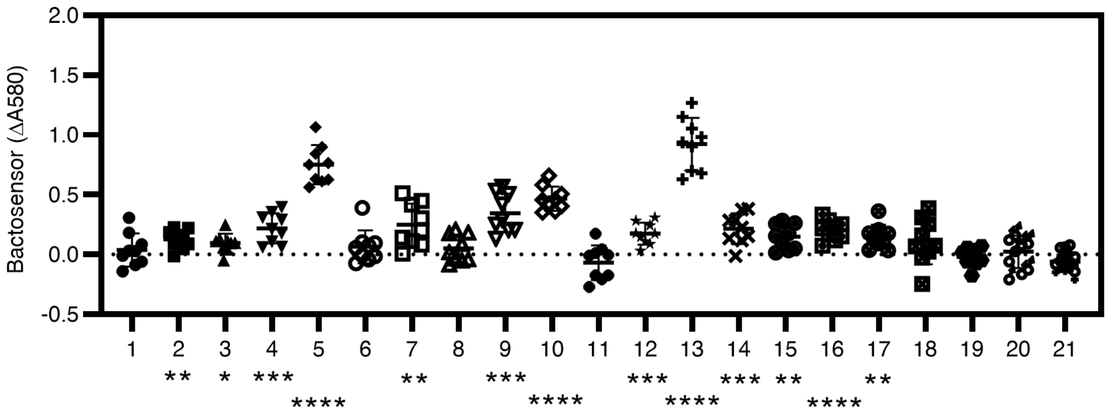

Supplementary Figure 18: Statistical analysis of TcpP-LacZ in 21 clinical serum samples. The signal outputs of TcpP-LacZ to 21 clinical serum samples with significant differences (compared with the signal to serum from healthy donor) verified by unpaired two tailed student t-test are marked by asterisk (*p < 0.05, **p < 0.01, ***p<0.001, and ****p<0.0001, respectively). Complete data analysis detail (the exact P values) is available in the Source Data file. The data points correspond to the mean value of three replicates performed in triplicate on three different days (n = 3 biologically independent samples). Error bars: ± SD.

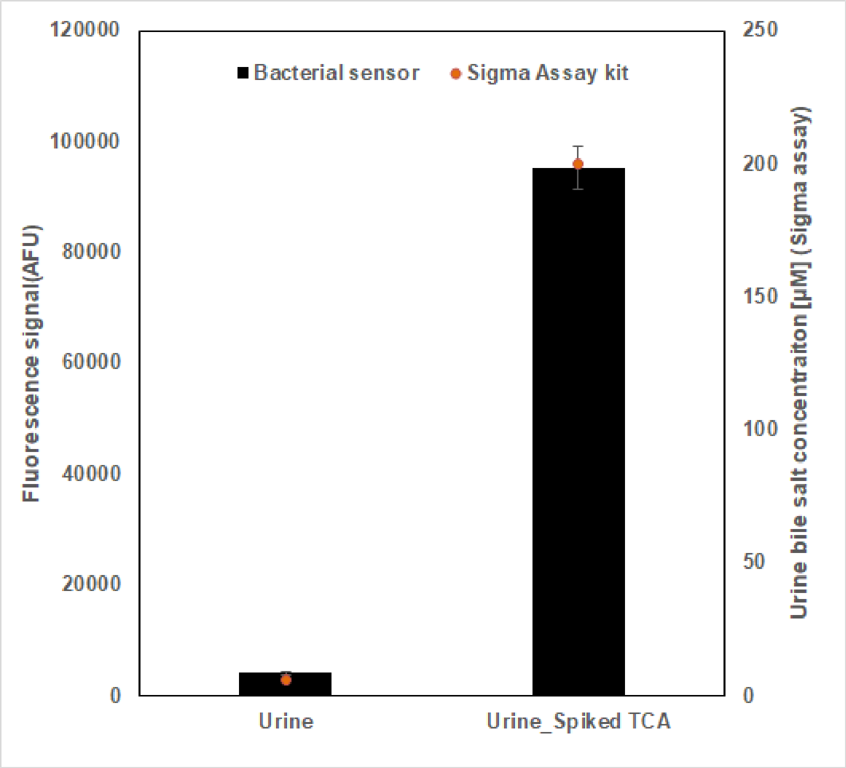

Supplementary Figure 19: The response of CadC-TcpP18 to spiked bile salts. Overnight cultures of cells with CadC-TcpP18 were diluted 1:100 into 1 mL of LB/chloramphenicol medium with 20% of urine spiked with 200 µM of TCA in 96 deep-well plates (Greiner bio-one), incubated at 37°C with vigorous shaking for further 16 hours and analyzed by flow cytometry. The bar graphs correspond to the mean value of three replicates. Error bars: ± SD.

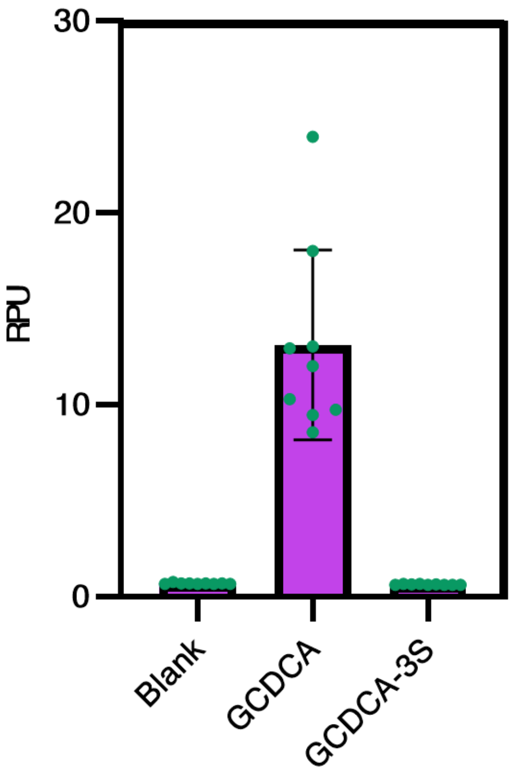

Supplementary Figure 20: Example of lack of response of CadC-TcpPH to sulfonated bile salts. The overnight cultures of cells with CadC-TcpP were diluted 1:100 into 1 mL of LB/chloramphenicol medium with 20 μM of GCDCA or GCDCA-3S (GCDCA sulfated at 3-OH position) in 96 deep-well plates (Greiner bio-one), incubated at 37°C with vigorous shaking for further 16 hours and analyzed by flow cytometry. Experiments were performed in triplicate. The result indicated that the sulfonation of GCDCA interferes with bile salt detection by CadC-TcpP. The bar graphs correspond to the mean value of three replicates performed in triplicate on three different days (n = 3 biologically independent samples). Error bars: ± SD. RPU: reference promoter units.

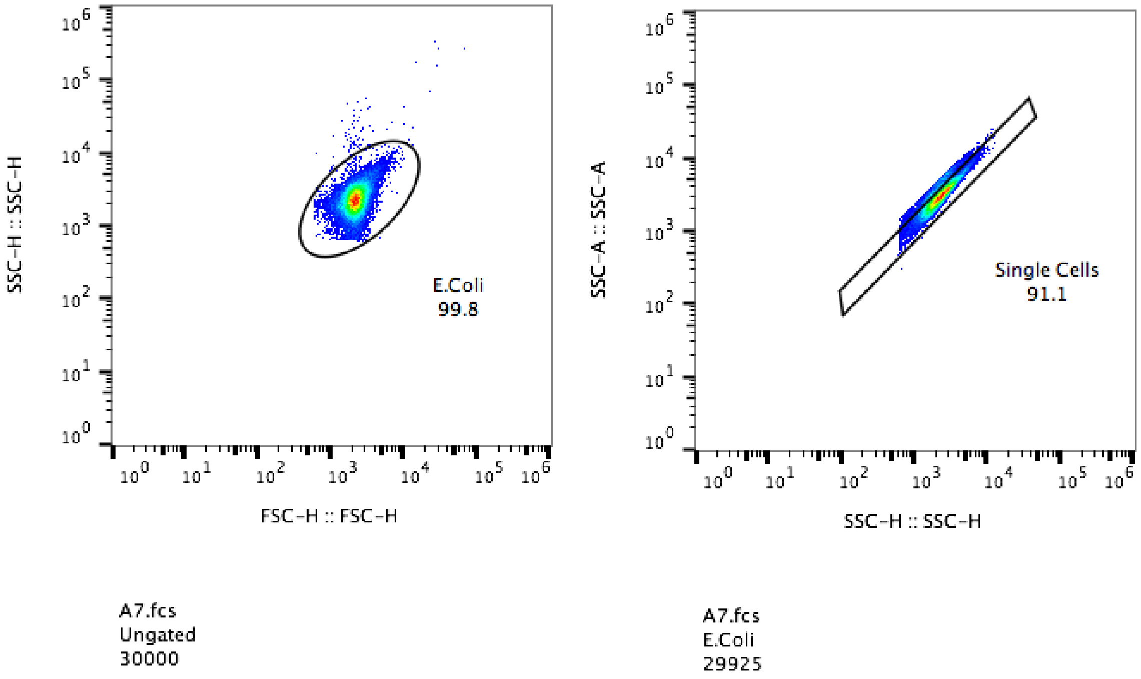

Supplementary Figure 21: Figure exemplifying the flow cytometry gate strategy. The *E.coli* strain containing constitutively expressed sfGFP was used as an indicator for the identification of living bacteria cells. Gates were designed based on FSC-H vs SSC-H graphs to remove debris from the analysis (left pannel) and SSC-A vs SSC-H to doublet discrimination (right panel).

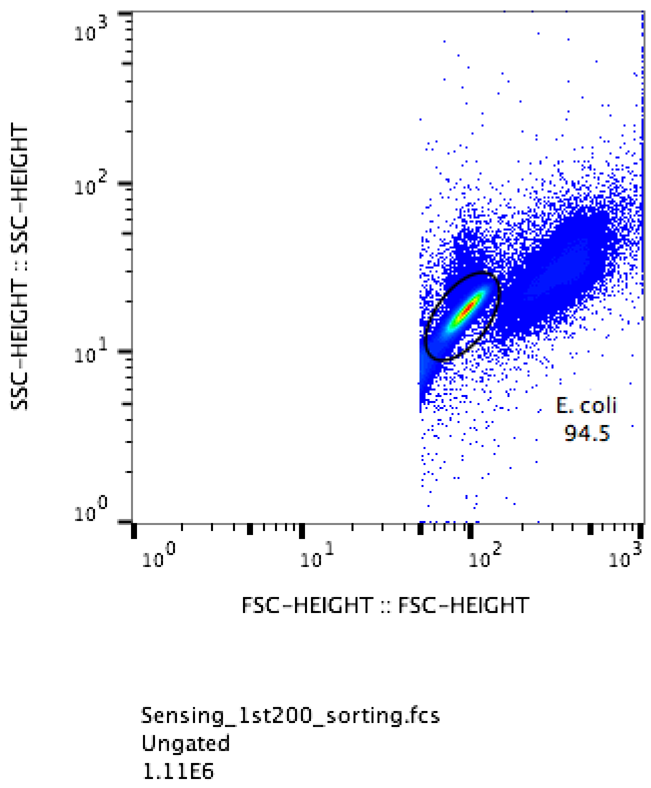

Supplementary Figure 22: Figure exemplifying the cell sorter gate strategy. The *E.coli* strain containing constitutively expressed sfGFP was used as an indicator for the identification of living bacteria cells. Gates were designed based on FSC-H vs SSC-H graphs to remove debris from the analysis. The abundance of relevant cell populations within post-sort fractions is about 94.5%.

Table S1 Genetic parts

Component	Nucleotide sequences
CadC DNA binding domain and juxtamembrane linker	ATGCAACAACCTGTAGTTCGCGTTGGCGAATGGCTTGTTACTCCGTCCATAAACCAAATTAGCCGCAATGGGCGTCAACT<br>TACCCTTGAGCCGAGATTAATCGATCTTCTGGTTTTCTTTGCTCAACACAGTGGCGAAGTACTTAGCAGGGATGAACTTATC<br>GATAATGTCTGGAAGAGAAGTATTGTCACCAATCACGTTGTGACGCAGAGTATCTCAGAACTACGTAAGTCATTAAAAGATA<br>ATGATGAAGATAGTCCTGTCTATATCGCTACTGTACCAAAGCGCGGCTATAAATTAATGGTGCCGGTTATCTGGTACAGCGA<br>AGAAGAGGGAGAGGAAATAATGCTATCTTCGCCTCCCCCTATACCAGAGGCGGTTCCTGCCACAGATTCTCCCTCCCAC<br>AGTCTTAACATTCAAAACACCGCAACGCCACCTGAACAATCCCCAGTTAAAAGCAAACGA
TcpP transmembrane domain and periplasmic domain	GTGGTGCCGTATCTGGTGTTTTCAGCGTTATATGTTGCCTTATTACCTGTCATTTGGTGGTCATACGGGCAATGGTATCAACA<br>CGAATTGGCCGGGATTACTCACGATTTACGTGACTTAGCCCGTCTTCCTGGAATCACAATCCAGAAACTTAGCGAGCAGA<br>AGTTGACCTTTGCTATCGACCAGCATCAATGTTCGGTGAATTACGAACAAAAGACGTTGGAATGTACTAAGAACTAATAA
TcpH	ATGCACAAGAAGTTAAAGGCTTGGGGAGGGGCCACTGGTCTGTTTGTGGTCGCCCTGGGGGTAACAATTATTGCTCTTC<br>CTATGCGCCAGAAAAATAGCCACGGGACCATGATTATTGATGGAACCGTTACTCAAATCTTTAGCACCTACCAGGGCAATC<br>TGTCTAATGTGTGGCTTACTCAGACGGACCCTCAAGGTAATGTTGTTAAATCATGGACTACACGCTACCAAACATTGCCTGA<br>CCCTTCATCACAAAAGCTGAATCTGATCCCCGATTACTCCCAATCGAATGCTTCTCGTGATTACAATGTATTGTCGATCTATC<br>AACTTGGCAAAGGGTGCTTTCTGGCTTTTCCTTACAAATTGCTGACGGCGGAAAAGATGTGGTTCTCGTGTCAGAGCGAT<br>TTCTAA
VtrA transmembrane domain and periplasmic domain	ATTCTTCTTATCAGCATTATTTTGCAGTTGGCTTTCATCATCTATGTGGCTTATAGCTACACCAGTATTTTCGTGTCGTCCACC<br>GCAAAGGACGACTATCCCAGTTTATCGTTCCAACAAGACTACGTCTACATCTTTTCTTCAGACTTTCAATTAAGCGAGGAG<br>CTGGGCGTGGCCCTTATCAACGCTTTAAGTGCAAAAGAAATTGTACCCGAGCGTCTTTACGTTATGTTGAATGACAAGACC<br>ATCTCGTTCAGTTTTATCAGCAAGAATAAGAAGTCCAAGAATCGCGTATTAAGCACGGAAAAGAAACTCAATTATAAGCATAT<br>CTCCGAGTACATCGTCAATGAGATTGAGTACTAATAA
VtrC	ATGAAACTTAATATTAAGCGTCTGCATTTGAGCCTGACCCTGATGAGTGTCGTCATGCTGCTGGTAATCATCTACAATAATTT<br>CTTTCAACCCGTGCACTTCTACGAAACGTCATACAAGTACCAAGCGGCGGACTCGACGTACATGCACGACGTCGCGATC<br>AATGTCTCGATTAAGGGCAACCACTTCACCTCAGATATCATCATCCGCGAGCTGGTGAAGTCAGAGAACAAGAATTACTAT<br>AACGTTATTGGACATGGAGACATCATTCAAAAGAACACCCATCAATACTACTTGAACTTCGATAACATCGACGTTTACACGG<br>GTACTAATAAAGCGAACATGAAGCCGTATAAGGAACCGACTAGCATCTCCTCGCTCATCAACAAGTCAAATAACATTCGCG<br>TTGTTTACTTATCGGAAGAGTATGTTGTGGTAGAGTTCTTCTTTTATGATGGACAGATTATCACATTGCATCGCTATTAATAA
sfGFP	ATGTCAAAAGGAGAAGAACTTTTTACAGGTGTAGTACCTATCTTGGTTGAATTGGATGGTGATGTTAACGGTCACAAATTTT<br>CTGTACGTGGTGAAGGTGAAGGTGATGCAACTAACGGTAAATTGACACTTAAATTCATTTGTACAACTGGAAAACTTCCTGT<br>TCCTTGGCCTACTCTTGTTACAACATTGACATATGGAGTACAATGTTTTTCACGTTATCCTGATCATATGAAACGTCACGATTT<br>TTTTAAATCTGCTATGCCAGAAGGTTATGTACAAGAACGTACAATTTCATTTAAAGATGACGGAACATATAAAACACGTGCTG<br>AAGTAAAATTCGAAGGTGACACTCTTGTTAATCGTATCGAATTGAAAGGAATCGATTTCAAAGAAGATGGTAACATTTTGGG<br>ACACAAACTTGAATACAACTTCAACTCTCATAATGTTTATATCACAGCTGACAAACAAAAAAACGGTATTAAAGCTAATTTTAA<br>AATTCGTCACAATGTTGAAGATGGATCTGTTCAATTGGCTGATCATTATCAACAAAATACACCAATCGGAGACGGACCAGTA<br>TTGCTTCCAGATAACCACTACCTTTCTACTCAATCAGTTCTTTCAAAAGATCCTAACGAAAAACGTGACCATATGGTACTTCT<br>TGAATTTGTTACAGCAGCAGGTATCACTCACGGTATGGACGAACTTTATAAATAA
**Promoters**	**Nucleotide sequences**
P14	TTGACAATTAATCATCCGGCTCGTATAATGTGTGGA
P10	TTTCAATTTAATCATCCGGCTCGTATAATGTGTGGA
P9	TTGCCTCTTAATCATCGGCTCGTATAATGTGTGGA
pCadBA	ATCCATTGTAAACATTAAATGTTTATCTTTTCATGATATCAACTTGCGATCCTGATGTGTTAATAAAAAACCTCAAGTTCTCACT<br>TACAGAAACTTTTGTGTTATTTCACCTAATCTTTAGGATTAATCCTTTTTTCGTGAGTAATCTTATCGCCAGTTTGG

Table S2-5 Patient characteristics

Table S2. Patients characteristics (n=21)

Characteristics	Value
Age (years)	57.5±10.4
Male, n (%)	15 (71.4)
Underlying liver disease	
Alcoholic cirrhosis, n (%)	7(33.3)
Hepatocellular carcinoma, n (%)	4 (19)
Virus related cirrhosis (HBV, HCV), n (%)	2 (9.5)
Primary sclerosing cholangitis or primitive biliary cholangitis	5 (24)
Other (non-alcoholic steato-hepatitis, Budd-Chiari,autoimmune hepatitis)	3 (14.2)
Time from liver transplantation to serum analysis in months, median (SD)	8± 38
Still alive at the end of the study , n (%)	18 (85.7)

Table S3. Patients characteristics (n=21)

Patient number	Age range	Sex	Cause of transplant	Year of transplant
1	50-69	m	Alcoholic	2020
2	60-69	f	NASH	2019
3	60-69	m	HCC	2019
4	60-69	m	Alcoholic	2019
5	30-39	m	vascular cirrhosis	2020
6	60-69	m	HCV	2006
7	40-49	f	auto-immune acute hepatitis	2020
8	60-69	m	PSC	2020
9	60-69	f	cirrhosis NASH	2020
10	40-49	f	PSC	2014
11	60-69	f	autoimmune cirrhosis	2019
12	>70	m	budd chiari disorder	2019
13	60-69	m	alcoholic cirrhosis	2013
14	30-39	m	PSC	2019
15	>70	m	NASH	2019
16	60-69	m	ischemic cholangitis	2019
17	50-59	m	HVC/HCC/alcoholic cirrhosis	2019
18	50-59	f	HVC/HCC/alcoholic cirrhosis	2019
19	50-59	m	HBV	2018
20	60-69	f	PSC	2020
21	40-49	f	HBV/HCV/HDV	2016

PSC: Primary Sclerosing Cholangitis

HBV: Hepatitis B Virus

HCV: Hepatitis C Virus

HCC: Hepato Cellular Carcinoma

NASH: Non-Alcoholic SteatoHepatitis

Table S4. Patients characteristics (n=21)-Hepatic tests and bactosensor measurements

Patient number	ASAT	ALAT	GGT	Pal	Total bilirubin	hospital bile salt	Bile salt (sigma kit)	BacSensor (△OD 580)
1	13	7	18	76	10	4,7	2,56	0,04
2	66	33	214	212	12	24	34,44	0,13
3	25	11	24	81	5	7,5	7,59	0,10
4	23	33	94	78	11	5,4	7,86	0,22
5	168	256	96	181	186	193,1	166,21	0,75
6	27	98	959	160	13	2,7	0,62	0,06
7	20	11	136	159	13	19	76,22	0,25
8	10	10	36	127	20	7,9	7,86	0,05
9	40	13	154	187	16	25	38,74	0,35
10	55	32	60	222	30	55,8	72,91	0,47
11	26	17	14	61	3	10,4	18,24	-0,07
12	14	7	94	118	5	13,2	15,65	0,17
13	20	13	41	66	101	77	169,91	0,92
14	28	34	28	137	5	10,6	9,63	0,22
15	19	23	78	193	6	11,3	11,10	0,15
16	18	15	29	175	8	40,9	56,58	0,20
17	24	32	81	129	5	8,4	12,07	0,15
18	21	13	50	149	5	11,2	15,37	0,11
19	24	15	17	83	5	3,6	20,71	-0,02
20	13	13	28	103	17	12,6	20,08	0,02
21	31	72	143	88	6	3,2	12,60	-0,06

Table S5. Patients acute clinical conditions

Patient number	Acute clinical condition
1	follow up
2	bypass/switch duodéno pancréas
3	follow up
4	type 2 diabetes
5	transplant liver and kidney, graft rejection/ biliary stenosis
6	graft rejection and biliary duct obstruction/stenting 48 H before blood test
7	Kehr drain
8	follow up
9	sepsis (acute general infection) and hepatic artery stenosis
10	follow up and new cirrhosis
11	fiever
12	ischemic cholangitis
13	type 2 diabetes
14	follow up
15	follow up
16	follow up, phosphatase alcaline d'origine osseuse, portage BLSE urinaire
17	follow up and bile duct stenosis
18	follow up
19	acute colic infection
20	follow up
21	angiocholitis

Table S6 Comparison of different methods used for bile salt profiling in various diseases.

Condition	Detection method	Sample type	Alternation of bile acid	Note	Ref
Liver Obstruction	LC-MS/MS	100 µL serum/urine	Total serum bile salt concentration:<br>2.7 µM (Health) -><br>156.9 µM (Patient)	Primary conjugated bile acid concentration:<br>1.7 µM (Health) -><br>156.8 µM (Patient)	[30]
Cirrhosis	HPLC	50 mg freeze-dried stool	Total fecal bile salt amount:<br>206.5 µg/100mg (Health) -><br>39 µg/100mg dry stool (Advance cirrhosis)	Ratio of 2nd/1st bile salt:<br>79.8 (Health) -><br>0.004 (Advance cirrhosis)	[77]
Drug induced liver injury (acetaminophen overdose)	LC-MS/MS	50 µL serum	Total serum bile salt concentration:<br>4.8 µM (Health) -><br>110 µM (Patient)	Primary conjugated bile salt has the most significant fold change (30 - 80 fold) comparing to rest of the bile salt format	[32]
Patients with low or medium MELD score	LC-MS/MS		Total urine bile salt concentration:<br>9.1 µM (Health) -><br>71.3 µM (Patient)	Total urine sulfated bile salt concentration:<br>8.5 µM (Health) -><br>67.9 µM (Patient)	[78,79]

Table S7 Bile salt profile in different liver dysfunction (Liver obstruction and nonalcoholic steatohepatitis

(Unit: µM)	Control	Liver obstruction	NASH with cirrhosis	Patients with Low and medium MELD score	
Sample type	serum		serum	Urine (Focus on [sulfonated BA] here)	
CA	0.181	0.173	8.7	0.004	0.025
**GCA**	**0.233**	**56.22**	**54.9**	**0.053**	**1.262**
**TCA**	**0.179**	**54.48**	**18.5**	**0.141**	**1.376**
CDCA	0.256	0.084	15.3	0.073	0.229
**GCDCA**	**0.771**	**28.53**	**241.9**	**2.399**	**25.494**
**TCDCA**	**0.12**	**16.57**	**60.1**	**0.057**	**2.598**
UDCA	0.137	0.02	115.1	0.459	1.855
GUDCA	--	--	166.8	1.116	23.840
TUDCA	0.005	0.074	9	0.026	1.797
DCA	0.386	0.02	8	0.002	0.001
GDCA	0.246	0.417	25.9	2.991	6.011
TDCA	0.044	0.213	2.8	0.221	0.569
LCA	0.012	0.001	0.9	0.007	0.041
GLCA	0.016	0.01	2.2	0.808	2.463
TLCA	0.023	0.011	0.4	0.222	0.748
Ref	[30]		[80]	[78,79]	

- The method used in these two experiments are LC-MS/MS
- MELD score: Model for End-stage Liver Disease score
- --: the data is not shown in the paper

Table S8 Comparison of different bile salt assay methods

Method	Advantage	Disadvantage	Processing time	Ref
HPLC	High sensitivity and specificity, has good agreement with GC-MS and LC-MS/MS	Not suitable for conjugated bile salts (require deconjugation). Complicated sample treatment, long detection time which is not for large amount of samples	Sample preparation, especially deconjugation, will take more than 16 hours. Unable to process multiple samples in parallel.	[81,82]
GC-MS	High degree of separation giving accurate molecular information for non-conjugated BAs.	Not suitable for conjugated bile salts (high boiling points). Require sample pretreatment and long detection time	Sample preparation will take about 2 -3 hours. Unable to process multiple samples in parallel.	[81,83]
LC-MS/MS	Short analysis time, low detection limit, able to distinguish conformational isomers, and **most complete bile salt profile**. Simple sample treatment comparing to HPLC and GC-MS [81]	High cost, complicated instrumental operation, suit for basic research but not clinical use.	Sample preparation will take about 2 -3 hours. Unable to process multiple samples in parallel.	[81,84]
Antibody based ELISA	Easy for operation in lab, suit for large amount of samples	Low accuracy, antibodies usually have cross reactions with metabolites or matrices to produce false positive results.	Sample binding and substrate reaction for 1 - 2 hours. Able to process multiple samples in parallel.	[85]
Enzymatic method	Routine clinical detection method of TBA, simple operation (30 - 45 mins), low sample amount (5 -50 μL), and low cost (~ 45 euro / triplicate)	Can't detect bile acids with C3-OH modification (such as sulfonation in urine bile acids)	Able to detect bile salts in serum directly, 30 - 45 minutes reaction time, Able to process multiple samples in parallel.	[86]
Bacto-sensor	Targeting primary conjugated bile salts which are closely related to liver dysfunction. Relatively simple sample preprocessing compared to HPLC, GC-MS and LC-MS/MS. Low sample amount (5 μL), low cost (~ < 3 euro / triplicate), one-pot assay method without further washing or shaking, suited for processing huge amount of samples at the same time, incubation can be performed at body temperature (e.g. armpit)	Requires pre-processing of serum samples (heat-inactivation), requires further process to avoid the interferences from clinical samples, (not as robust as enzymatic methods), requires further development for i. long term storage as lyophilization; ii. adapting for portable devices	Serum samples need to be heat-inactivated for 30 mins. Incubation and processing time for 2 - 3 hours. Able to process multiple samples in parallel.	This study

Table S9 Primers

For 4 NNK library construction	
Vector preparation	
V5	GGTCTCAGACCACTTCCGAGTAGAATCG
V3	GGTCTCAGCTGGTCGATAGCAAAGGTCAAC
Insert preparation	
I5	GGTCTCACAGCATCAATGTTCGGTGNNKNNKNNKNNKAAGACGTTGGAATGTACTAAGAACTAATAAAC
I3	GGTCTCAGGTCAATAA TACCGAAACTATTTTATTGTTC

**For NGS sequencing**	
TcpP_Sensing_NGS_1st_Fw_20200220	TCGTCGGCAGCGTCagatgtgtataagagacagNNNNNNNNCCAGAAACTTAGCGAGCAGAAG
TcpP_Sensing_NGS_1st_Rv	GTCTCGTGGGCTCGGagatgtgtataagagacagNNNNNNNNgccacctgggatttccg

SUPPLEMENTARY REFERENCES

77. Kakiyama, G. *et al.* Modulation of the fecal bile acid profile by gut microbiota in cirrhosis. *J. Hepatol.* **58**, 949–955 (2013).

78. Bathena, S. P. R., Mukherjee, S., Olivera, M. & Alnouti, Y. The profile of bile acids and their sulfate metabolites in human urine and serum. *J. Chromatogr. B Analyt. Technol. Biomed. Life Sci.* **942-943**, 53–62 (2013).

79. Bathena, S. P. R. *et al.* Urinary bile acids as biomarkers for liver diseases II. Signature profiles in patients. *Toxicol. Sci.* **143**, 308–318 (2015).

80. Sydor, S. *et al.* Altered Microbiota Diversity and Bile Acid Signaling in Cirrhotic and Noncirrhotic NASH-HCC. *Clinical and Translational Gastroenterology* vol. 11 e00131 (2020).

81. Kakiyama, G. *et al.* A simple and accurate HPLC method for fecal bile acid profile in healthy and cirrhotic subjects: validation by GC-MS and LC-MS. *J. Lipid Res.* **55**, 978–990 (2014).

82. Shi, Y. *et al.* Simultaneous quantification of the major bile acids in Artificial Calculus bovis by high-performance liquid chromatography with precolumn derivatization and its application in quality control. *Journal of Separation Science* vol. 38 2753–2762 (2015).

83. Tsai, S.-J. J., Zhong, Y.-S., Weng, J.-F., Huang, H.-H. & Hsieh, P.-Y. Determination of bile acids in pig liver, pig kidney and bovine liver by gas chromatography-chemical ionization tandem mass spectrometry with total ion chromatograms and extraction ion chromatograms. *J. Chromatogr. A* **1218**, 524–533 (2011).

84. John, C. *et al.* A liquid chromatography-tandem mass spectrometry-based method for the simultaneous determination of hydroxy sterols and bile acids. *J. Chromatogr. A* **1371**, 184–195 (2014).

85. Baldofski, S. *et al.* Enzyme-linked immunosorbent assay (ELISA) for the anthropogenic

marker isolithocholic acid in water. *J. Environ. Manage.* **182**, 612–619 (2016).

86. Griffiths, W. J. & Sjövall, J. Bile acids: analysis in biological fluids and tissues. *J. Lipid Res.* **51**, 23–41 (2010).
