# Supplementary material for: Programmable receptors enable bacterial biosensors to detect pathological biomarkers in clinical samples
Source: Nat Commun. 2021 Sep 1;12:5216. doi: 10.1038/s41467-021-25538-y (PMC8410942; doi:10.1038/s41467-021-25538-y)
Supplement: Supplementary file 2 — Reporting Summary [file 41467_2021_25538_MOESM2_ESM.pdf]

nature research

Corresponding author(s): Jerome Bonnet
Last updated by author(s): Aug 6, 2021

Reporting Summary

Nature Research wishes to improve the reproducibility of the work that we publish. This form provides structure for consistency and transparency in reporting. For further information on Nature Research policies, see our Editorial Policies and the Editorial Policy Checklist.

Statistics

For all statistical analyses, confirm that the following items are present in the figure legend, table legend, main text, or Methods section.

n/a	Confirmed	
☐	☒	The exact sample size (*n*) for each experimental group/condition, given as a discrete number and unit of measurement
☐	☒	A statement on whether measurements were taken from distinct samples or whether the same sample was measured repeatedly
☐	☒	The statistical test(s) used AND whether they are one- or two-sided<br>*Only common tests should be described solely by name; describe more complex techniques in the Methods section.*
☒	☐	A description of all covariates tested
☐	☒	A description of any assumptions or corrections, such as tests of normality and adjustment for multiple comparisons
☐	☒	A full description of the statistical parameters including central tendency (e.g. means) or other basic estimates (e.g. regression coefficient) AND variation (e.g. standard deviation) or associated estimates of uncertainty (e.g. confidence intervals)
☐	☒	For null hypothesis testing, the test statistic (e.g. *F*, *t*, *r*) with confidence intervals, effect sizes, degrees of freedom and *P* value noted<br>*Give P values as exact values whenever suitable.*
☒	☐	For Bayesian analysis, information on the choice of priors and Markov chain Monte Carlo settings
☒	☐	For hierarchical and complex designs, identification of the appropriate level for tests and full reporting of outcomes
☒	☐	Estimates of effect sizes (e.g. Cohen's *d*, Pearson's *r*), indicating how they were calculated

Our web collection on statistics for biologists contains articles on many of the points above.

Software and code

Policy information about availability of computer code

Data collection	Flow cytometry acquisition performed using Attune NxT software version 2.7. Cell sorting performed using Biorad S3 software Prosort version 1.6. Plate reader performed using Gen5 Microplate Reader and Imager Software version 3.03.
Data analysis	Python script (in Python 3.6) for NGS reads calculator is available from Github Page: https://github.com/hungjuchang/NGS-Sequence-Counts_PWM_PSSM-calculator.git. The sequence logo and enrichment score analysis is through Rscript Logolas version 1.3.1, which is available from https://github.com/kkdey/Logolas. Flow cytometry data analysis performed using Flowjo 10.0.8r1 (treestar). Statistical analysis performed using GraphPad Prism 8.0.2.

For manuscripts utilizing custom algorithms or software that are central to the research but not yet described in published literature, software must be made available to editors and reviewers. We strongly encourage code deposition in a community repository (e.g. GitHub). See the Nature Research guidelines for submitting code & software for further information.

Data

Policy information about availability of data

All manuscripts must include a data availability statement. This statement should provide the following information, where applicable:

- Accession codes, unique identifiers, or web links for publicly available datasets
- A list of figures that have associated raw data
- A description of any restrictions on data availability

Data availability
The authors declare that all data supporting the findings of this study are available within the paper, its supplementary information files, or public repositories. NGS data have been deposited in NCBI SRA database with the BioProject ID: PRJNA714981 (https://www.ncbi.nlm.nih.gov/bioproject/714981). The flow cytometry data have been deposited in FlowRepository (https://flowrepository.org), with IDs: FR-FCM-Z3K6 (Fig.2b), FR-FCM-Z3KL (Fig.2d), FR-FCM-Z3KM (Fig.2e), FR-FCM-Z3KT (Fig.3e_V3_7_11), FR-FCM-Z3KU (Fig.3e_V14_16_18), FR-FCM-Z3KV (Fig.3e_V19_22_78), FR-FCM-Z3KR (Fig.S3A), and FR-FCM-Z3LZ (Fig.S3B). Source data are provided with this paper.

Field-specific reporting

Please select the one below that is the best fit for your research. If you are not sure, read the appropriate sections before making your selection.

☒ Life sciences ☐ Behavioural & social sciences ☐ Ecological, evolutionary & environmental sciences

For a reference copy of the document with all sections, see nature.com/documents/nr-reporting-summary-flat.pdf

Life sciences study design

All studies must disclose on these points even when the disclosure is negative.

Sample size	For NGS data, maximum 1.4 x 10^3 different variants were sorted at the first round of cell sorting (1.4 % of cell population from 10^5 cells sorted). With the sample size ~ 10^7 sequencing reads provided by Novogen illumina MiSeq PE250, each variant has ~ 1,000 reads for further analysis. Rest of the experiments other than those involving NGS data, were performed in triplicate on separate days, with three technical replicates each. This was enough to determine normality, spread of the data and provide valid statistical analysis.
Data exclusions	No data exclusion only for NGS accroding to the following methodology: Paired-end reads were merged without quality filtering using NGmerge algorithm, allowing dovetailing. Reads that were not successfully merged were rejected from further analysis.<br>Sequences where then mapped to reference sequences using BWA-mem.<br>Reads that did not map were rejected from further analysis. The exclusion criteria were pre-established.
Replication	All experiments are the mean of three experiments performed in triplicate on three different days. All attempts at replication were successful.
Randomization	For FACS collecting TcpP loop variants, 5 x 10^5 cells were randomly collected and analyzed by Bio-rad S3 sorter. For single colony analysis of library functional variants, 96 colonies were randomly picked and analyzed by flow-cytometry. Other experiments were performed to characterize the performances of DNA constructs, or their responses to indicated ligands. Therefore randomization was not needed in the design of these experiments.
Blinding	Experiments other than those involving analysis of clinical samples are characterizing the performances of designed constructs, or their responses to indicated ligands. Therefore blinding was not needed in the design of these experiments. For clinical sample analysis, experiments were single blinded. Samples were collected and their total bile salt concentration measured at the hospital. The samples were provided as blinded collection to the CBS, which performed testing using the bactosensor without knowledge of the bile salts measurements by enzymatic assay. The two types of measurements were then compared.

Reporting for specific materials, systems and methods

We require information from authors about some types of materials, experimental systems and methods used in many studies. Here, indicate whether each material, system or method listed is relevant to your study. If you are not sure if a list item applies to your research, read the appropriate section before selecting a response.

Materials & experimental systems

n/a	Involved in the study
☒	☐ Antibodies
☒	☐ Eukaryotic cell lines
☒	☐ Palaeontology and archaeology
☒	☐ Animals and other organisms
☐	☒ Human research participants
☒	☐ Clinical data
☒	☐ Dual use research of concern

Methods

n/a	Involved in the study
☒	☐ ChIP-seq
☐	☒ Flow cytometry
☒	☐ MRI-based neuroimaging

Human research participants

Policy information about studies involving human research participants

Population characteristics	The detail patient characteristics are available in the Table. S2 in the manuscript.
Recruitment	Patients were recruited among people that have been subjected to liver transplant between November 2006 and April 2020 and were visiting the Hepatology and Liver Transplantation Unit, Hôpital Saint-Eloi in Montpellier (France) for their regular follow-up medical visit. No other criteria besides having been transplanted for liver was applied, and patients samples were collected on a chronological order until reaching 21 patients.
Ethics oversight	The ethics committee of the University Hospital of Montpellier (Department of Hepatogastroenterology, Hepatology and Liver Transplantation Unit, Saint Eloi Hospital, University of Montpellier, Montpellier, France.) granted ethical approval (Number: 198711) and all patients signed an informed consent.

Note that full information on the approval of the study protocol must also be provided in the manuscript.

Flow Cytometry

Plots

Confirm that:

☒ The axis labels state the marker and fluorochrome used (e.g. CD4-FITC).

☒ The axis scales are clearly visible. Include numbers along axes only for bottom left plot of group (a 'group' is an analysis of identical markers).

☒ All plots are contour plots with outliers or pseudocolor plots.

☒ A numerical value for number of cells or percentage (with statistics) is provided.

Methodology

Sample preparation	Bacterial strain NEB10beta was purchased from NEB (New England Biolab). The induced bacterial cultures with different variants were diluted 100 folds to the concentration about 10^6 cells per mL before analysis.
Instrument	Attune NxT flow cytometer (Thermo Fisher) equipped with an autosampler
Software	Attune NxT™ Version 2.7 Software for acquisition, FlowJo for analysis.
Cell population abundance	For flow cytometry, about 90.9% of population were collected by using E.coli strain containing constitutively expressed sfGFP as an indicator for living bacteria cells, and doublet discrimination to remove the aggregated cells. For cell sorting, the E.coli strain containing constitutively expressed sfGFP was used as an indicator for the identification of living bacteria cells. Gates were designed based on FSC-H vs SSC-H graphs to remove debris from the analysis. The abundance of relevant cell populations within post-sort fractions is about 95%. The details about the abundance of relevant cell populations within the post-sort fraction are described in Fig.3d. In brief, 1.4%, 8.8% and 3.8% of cell populations were collected after first to third rounds of cell sorting.
Gating strategy	For flow cytometry (refer to Fig.S21), the E.coli strain containing constitutively expressed sfGFP was used as an indicator for the identification of living bacteria cells. Gates were designed based on FSC-H vs SSC-H graphs to remove debris from the analysis (left pannel) and SSC-A vs SSC-H to doublet discrimination (right panel).<br><br>For cell sorter (refer to Fig.S22), The E.coli strain containing constitutively expressed sfGFP was used as an indicator for the identification of living bacteria cells. Gates were designed based on FSC-H vs SSC-H graphs to remove debris from the analysis. The abundance of relevant cell populations within post-sort fractions is about 94.5%.

☒ Tick this box to confirm that a figure exemplifying the gating strategy is provided in the Supplementary Information.
