# Supplementary material for: Programmable receptors enable bacterial biosensors to detect pathological biomarkers in clinical samples
Source: Nat Commun. 2021 Sep 1;12:5216. doi: 10.1038/s41467-021-25538-y (PMC8410942; doi:10.1038/s41467-021-25538-y)
Supplement: Supplementary file 5 — Description of Additional Supplementary Files [file 41467_2021_25538_MOESM5_ESM.pdf]

Title: Supplementary data 1

Description: NGS data of TcpP functional variants. NGS sequence counts of TcpP variants from preselected library and selected functional variants are listed in the supplementary file. The number labeled at the end of protein sequences refer to different NNK DNA sequences for the same amino acid composition. For example: GGGG1 = "ggg ggg ggg ggg" and GGGG2 = "ggg ggg ggg ggt"
